# Understanding hand hygiene adherence in neonatology: a qualitative study of behavioral determinants

**DOI:** 10.1017/ice.2025.82

**Published:** 2025-07

**Authors:** Tamara C. Bopp, Yvonne Strässle, Colette Wyler, Marie-Theres Meier, Lauren Clack, Walter Zingg, Jehudith R. Fontijn, Aline Wolfensberger

**Affiliations:** 1 Department of Infectious Diseases and Hospital Epidemiology, University Hospital Zurich and University of Zurich, Zurich, Switzerland; 2 Department of Neonatology, University Hospital Zurich and University of Zurich, Zurich, Switzerland; 3 Institute for Implementation Science in Health Care, University of Zurich Faculty of Medicine, Zurich, Switzerland

## Abstract

**Background:**

Hand hygiene is effective to prevent transmission of pathogens and healthcare-associated infections. Despite efforts by hospitals to improve hand hygiene adherence among healthcare practitioners (HCP), adherence in neonatology wards is often limited.

**Objective:**

Identifying determinants, i.e., facilitators and barriers, to hand hygiene adherence among frontline HCP in neonatology.

**Design:**

Qualitative implementation research study.

**Setting:**

Department of Neonatology of the University Hospital Zurich, Switzerland.

**Methods:**

Semi-structured interviews with frontline HCP and Infection Prevention and Control (IPC) experts were conducted in November 2022. Interviews were coded deductively according to the Theoretical Domains Framework (TDF) and the Capability, Opportunity, Motivation and Behavior model (COM-B), and inductively to capture nuances in the data. Determinants whose addressing was perceived to likely improve hand hygiene adherence in the current setting were rated as “high priority”.

**Results:**

A total of 42 interviews were conducted, 27 (64%) with nurses, six (14%) with physicians, four (10%) with other professions, and five (12%) with IPC experts. Sixteen determinants were identified, twelve of which were high-priority, four in each COM-B domain. Knowledge, attention control, planning workflows, and habits & automatisms were found in “Capability,” workload & emergencies, invisibility of germs, role models, and being observed in “Opportunity,” and bad conscience, experience consequences of (non-) adherence, self-reflection, and intention to adhere to hand hygiene in “Motivation.”

**Discussion/Conclusion:**

Facilitators from all COM-B domains and barriers from “Capability” and “Opportunity” influence hand hygiene behavior in neonatology settings. Our findings can now inform interventions to improving hand hygiene adherence in neonatal settings.

## Introduction

Hand hygiene is a fundamental component of standard precautions in healthcare settings, helping to prevent the transmission of pathogens and healthcare-associated infections.^1^ In neonatal intensive care units (NICU), where patients are particularly susceptible to healthcare-associated infections due to their immature immune system, high adherence to hand hygiene is essential. Despite the strong evidence of hand hygiene effectiveness, adherence was reported to be inadequate in NICUs.^2–5^ For example, Lambe et al. reported an average adherence rate of 67% in 12 NICUs.^4^ Hand hygiene adherence in the neonatology department of the University Hospital Zurich (USZ) was also found to be improvable. Like other neonatology wards, the USZ neonatology has experienced outbreaks of pathogenic bacteria.^6–8^ Over the past years, inspired by the World Health Organization’s (WHO) multimodal hand hygiene improvement strategy, several interventions to increase hand hygiene adherence have been implemented.^1^ Despite significant resource investment, hand hygiene adherence still had room for improvement, healthcare practitioners (HCP) were repetitively observed to be unfamiliar with hand hygiene indications and technique, and the transmission of pathogens continued.

Hand hygiene adherence as per WHO indications and rubbing technique are driven by the individual behavior of HCP. Several theories, models and frameworks aim to help explain individual behaviors. Two of these models are the Capability, Opportunity, Motivation and Behavior model (COM-B),^9^ and the more granular Theoretical Domains Framework (TDF).^10,11^ Understanding the drivers and influences of individual behavior can inform the development of interventions specifically tailored to address identified barriers and facilitators.^12^ The Behavior Change Wheel with the COM-B model as its central component can guide and facilitate tailoring interventions.

While work has been done to investigate the drivers of individual behavior on hand hygiene in adult ICUs, little is known on such determinants in neonatal settings.^13^ Patient populations, though, are considerably different between NICU and adult ICU settings, which could affect risk perception. Settings vary in terms of equipment (e.g. incubators) and ongoing presence of family members. A study found that nurses in NICU (in comparison to adult ICU) spent more time physically caring for patients, but less time using monitors and devices.^14^ Frequent alarms occur as patients often experience apnea and hypotonia/bradycardia episodes, requiring physical stimulation to stabilize them.^15^ These factors affect hand hygiene indications and likely influence the underlying determinants of hand hygiene adherence. This study therefore seeks to describe the specific drivers of hand hygiene behavior in the NICU with the aim to inform the development of tailored hand hygiene interventions in this setting.

## Methods

### Study setting

The study was conducted in November 2022 in the Department of Neonatology of the USZ, Switzerland. The department has both a NICU and an intermediate care unit with a total of 32 places. The work force includes 113 nurses, 26 physicians, and four members of other professional groups (e.g., physiotherapists, and music therapists). The local neonatology infection prevention and control (IPC) team is supported by the hospital IPC team.

### Hand hygiene indications, technique, monitoring, and past interventions

The USZ neonatology ward follows the institutional concept of the “four moments for hand hygiene,” which is an adapted version of the “WHO my five moments for hand hygiene” ^1^ (Appendix 1). Specific to the neonatology setting, HCPs disinfect the forearms in addition to hands before and after contact with the patient. Hand disinfectant is provided by mounted dispensers (a minimum of one dispenser at every patient’s bedside, entrance, sink, dressing and intubation trolley, and ward round trolley), and wearable pocket dispensers. Hand hygiene is monitored through unannounced observations and monitoring of hand disinfectant consumption.

During the time of the interviews and in the preceding years, a number of interventions were carried out with the aim to improve hand hygiene adherence. These included, e.g., educational sessions in various forms, the use of a UV-Box with fluorescent hand disinfectant to train hand hygiene technique, institutionalization of peer feedback, and observation and feedback from hospital IPC experts and local IPC team.

### Theoretical frameworks

We used the TDF with 14 theoretical domains, and the COM-B model with its higher-level structure as theory-based guiding frameworks for this study. For a detailed description of the two frameworks and their components, please refer to Appendix 2.

### Study participants and data collection

All frontline HCP employed on the neonatology ward and all IPC experts (including the local neonatology IPC team and the hospital IPC experts responsible for the neonatology unit) were invited to participate in the study as interviewees. The HCPs were purposefully selected to ensure a representation of different professional groups, ages, genders, and years of work experience, ensuring a broad experience and knowledge. Interview participation was voluntary. The interviewees were informed about the reason for data collection (i.e., the planned tailoring of implementation strategies to increase hand hygiene adherence) and de-identification of collected data before analysis. Written informed consent from interviewees was obtained.

The interview guide of the semi-structured interviews was developed based on the TDF, pilot tested and refined based on feedback to ensure comprehension and coherence (see Appendix 3 for questionnaire). The IPC experts were asked to take a third-person perspective and report on the probable influences of hand hygiene adherence of the frontline staff.

Interviews took place on the neonatology ward and were conducted by a master medical student (TB), who was neither part of the department of neonatology, nor the hospital IPC team, and who received an in-depth training before conducting the first interview. The interviewer kept the discussion focused to the target behavior (i.e., hand hygiene adherence to indications and technique) and was committed to maintain an atmosphere of mutual trust and respect throughout the conversation. The interviews were audio recorded and transcribed verbatim.

### Data analysis

Two researchers (TB, AW) conducted coding independently. The researchers maintained a high level of reflexivity throughout the data analysis process,^16^ continuously reflecting on their own perspectives to ensure the integrity and credibility of the findings. Coded segments were compared, and, in case of disagreement, consensus was reached by discussion, or by consultation of a third researcher (LC).

The interview transcripts were first coded deductively by assigning relevant segments to the 14 TDF domains (with two separate domains “intentions” and “goals” merged to one domain “intentions and goals”) and identifying each as either a barrier or a facilitator. At the TDF level, the frequency of codes was calculated both for barriers and facilitators and compared between professional groups using a two-sample test of proportions.

Then, an inductive thematic analysis was conducted to capture nuances in the data and build more granular and context-specific categories. Finally, determinant themes (hereafter: determinants) were created across TDF domains, but within COM-B domains. “High priority” determinants were defined as factors whose addressing as barriers or leveraging as facilitators was likely to improve hand hygiene adherence in a quality improvement intervention in the current setting, in comparison to non-priority determinants which did not require modification. Last, relationships between determinants were described based on interview segments that showed positive or negative influences among them.

No specific coding software was used but the commentary function of Microsoft Word and Microsoft Excel to organize the coded segments. Statistical analyses were conducted with Stata 16.1^17^.

### Ethics

This context analysis was part of a quality improvement project, and formal ethical evaluation was waived by the Cantonal Ethics Commission (Req-2022-01325).

## Results

A total of 42 interviews were conducted, 27 (64%) with nurses, six (14%) with physicians, four (10%) with other professions, and five (12%) with IPC experts. Table 1 informs about the demographics of the interview participants. Median duration of the interviews was 21min (IQR 17 min–29 min).

**Table 1.** Summarizing the characteristics of the interview participants

Participant characteristic	Number n (%)
**Profession**	
Nurses	27 (64.3)
Physicians	6 (14.3)
Other professional groups	4 (9.5)
IPC experts	5 (11.9)
**Age groups (years)**	
21–24	6 (14.3)
25–29	11 (26.2)
30–39	7 (16.7)
40–49	8 (19.0)
≥50	10 (23.8)
**Gender**	
Female	40 (95.2)
Male	2 (4.8)
**Work experience on neonatology (years)**	
0–1	12 (28.6)
>1–4	12 (28.6)
>4–9	10 (23.8)
>9	8 (19.0)

*Abbreviations*: IPC, Infection Prevention and Control.

### TDF barriers and facilitators

**Figure 1.**
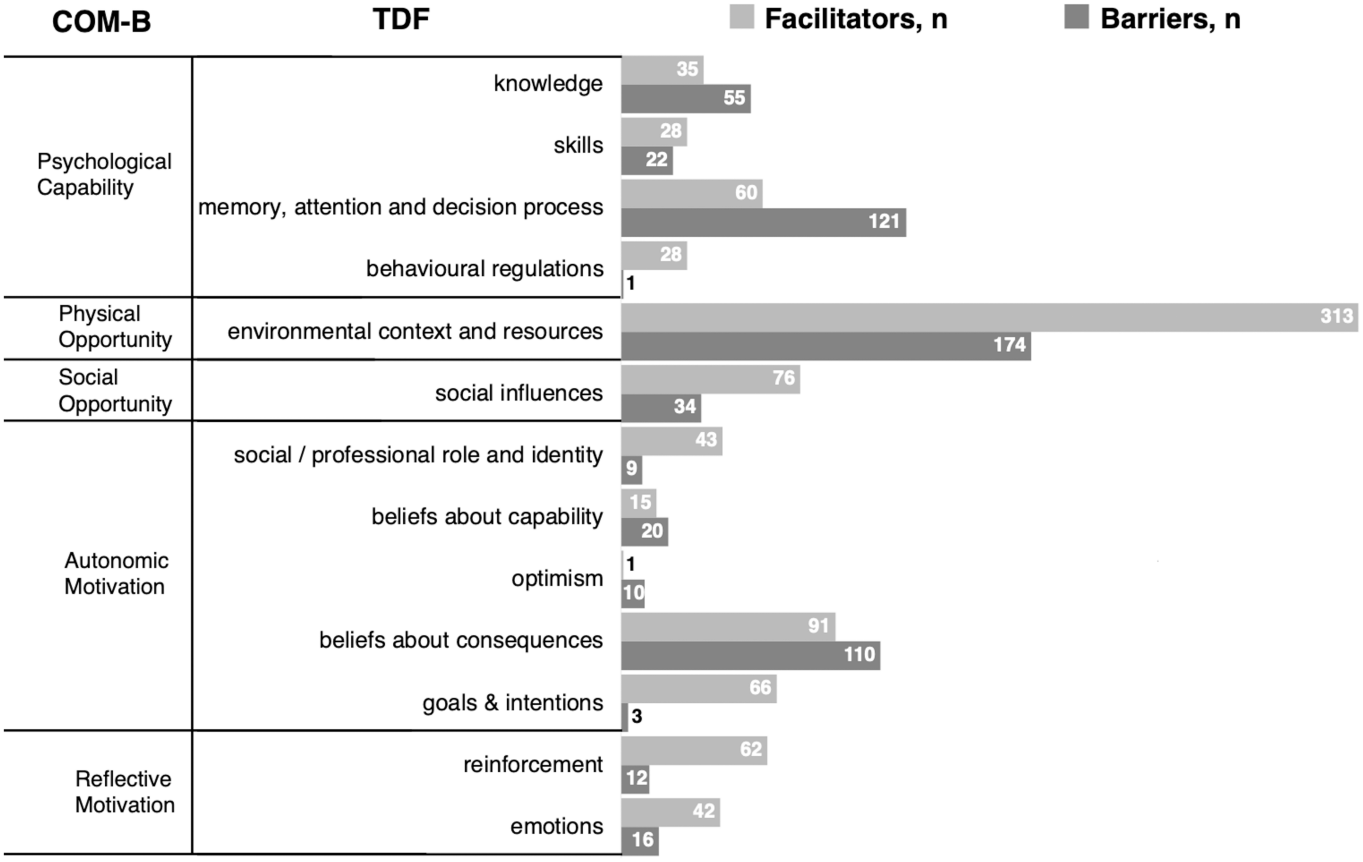
Frequency of barriers and facilitators according to the TDF and mapped to the COM-B model. Number of interview segments assigned to the specific domains of the TDF^10,11^ and COM-B,^9^ grouped in barriers and facilitators. *Abbreviations*: “COM-B”, Capability, Opportunity, Motivation and Behavior model; “TDF”, Theoretical domains framework.

A total of 1173 interview segments were coded and 1447 TDF codes were assigned, with 860 (59%) identified as facilitators, and 587 (41%) as barriers. The TDF domains most commonly coded as facilitators were “environmental context and resources,” “beliefs about consequences,” and “social influences.” Together, they represented more than half of the facilitators. The TDF domains most coded as barriers were “environmental context and resources,” “memory, attention and decision process” and “beliefs about consequences.” Together, they represented two-thirds of all barriers (Figure 1 and Appendix 4). Two of the three most mentioned determinants were consistent across the four professional groups, but IPC experts less often mentioned “beliefs about consequences” than the three other groups (each *P* < 0.01) (Appendix 5).

### Determinants of hand hygiene adherence

**Figure 2.**
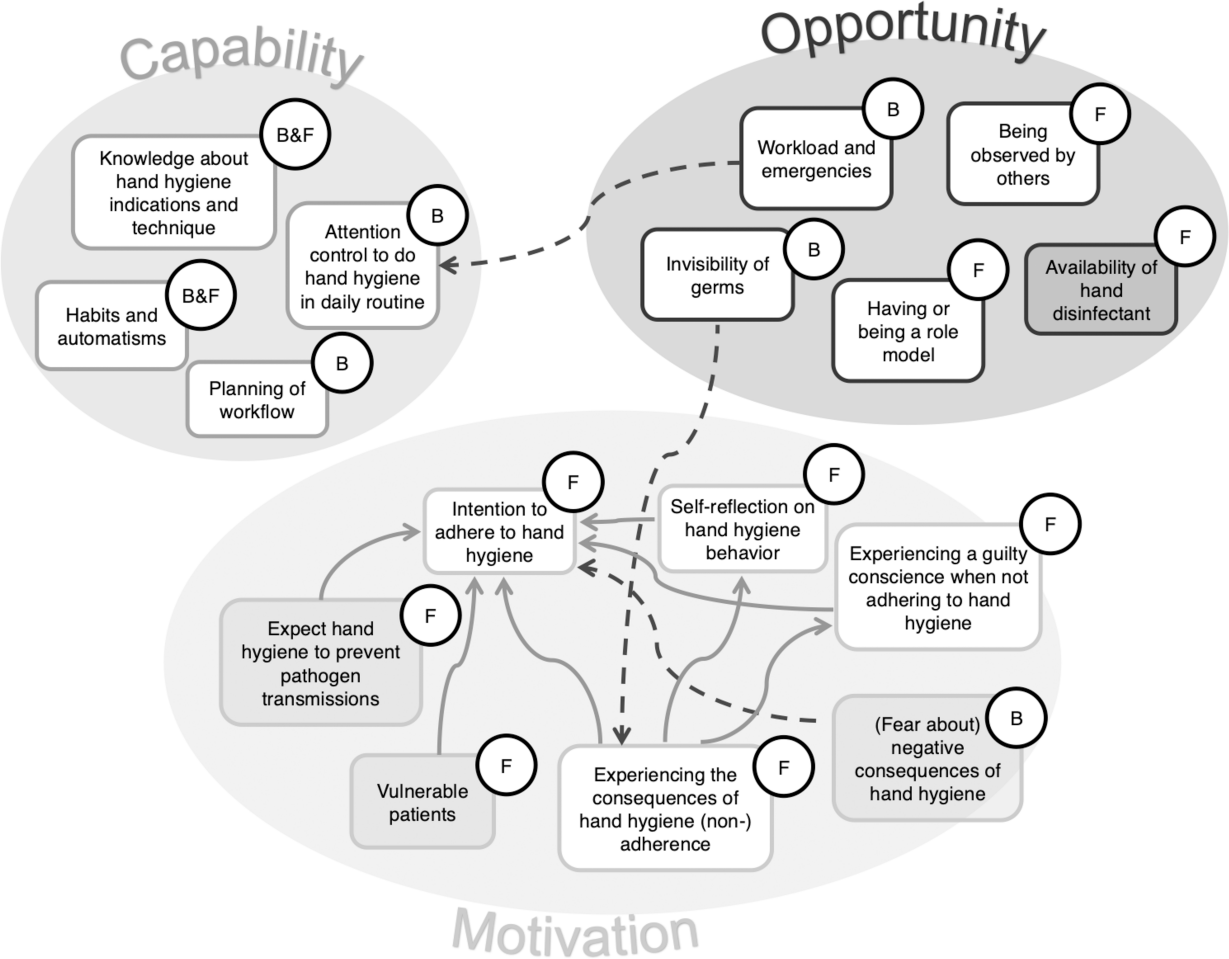
Determinants for hand hygiene adherence grouped by COM-B domains. All determinants are depicted in boxes with either white or gray background, grouped according to the three COM-B domains. Determinants in boxes with white background are high-priority, those with gray background are non-priority. The letters B and F indicate if the determinant was mostly mentioned to be present as a barrier (B) or a facilitator (F) for adherence. If Barriers and Facilitators both were mentioned equally, they were referred to as B&F. Solid arrows indicate positive interference, dashed arrows indicate negative interference between determinants. *Abbreviations*: “B”, barrier; “COM-B”, Capability, Opportunity, Motivation and Behavior model; “F”, facilitator.

Inductive thematic analysis led to identification of 16 determinants, twelve of which were deemed high priority, and four non-priority. Figure 2 visually summarizes the high-priority and non-priority determinants, the connections between them, and if they were mentioned mainly as facilitator, barrier, or both. Table 2 gives an in-depth overview and description, including illustrative quotes of interviewees.

**Table 2.** Summarizing the determinants themes (third column) identified in this study, mapped to the six COM-B domains (first column) and rated regarding priority (second column, X = high-priority). The fourth column describes the determinants in more detail, the fifth column provides some informative quotes from the interviews

COM-B Source of behavior	High priority	Determinant theme	Determinant description	Quote
Psycho-logical Capability	X	Knowledge about hand hygiene indications and technique	Knowledge was generally acknowledged to exist from the majority of HCP (facilitator), but many HCP revealed after targeted inquiry that they felt insecure regarding hand hygiene indications in specific moments and about the technique and indications of forearm disinfection (barrier). In situations of insecurity, HCPs often chose to perform hand hygiene, even when it might not be indicated. These self-appraisals by frontline staff were confirmed by the experts.	“(…) and regarding the forearm disinfection, as part of the new hand hygiene technique, I am not quite sure about how, where and what to do (…).” (nurse) “Yes, [I know the indications of hand hygiene] in theory.” (nurse)
	X	Attention control to do hand hygiene in daily routine	Lack of attention was one of the most frequently mentioned barriers, often attributed to external circumstances such as workload and emergencies.	“It [what prevents me from hand hygiene] is not thinking about it, being focused on something else, e.g., I have to set up the food, and then I just walk with the food [into the patient area]. And then I think: ‘OOPS, I should have disinfected my hands first.’ ” (nurse)
	X	Habits and automatisms	Habits and automatisms were mentioned to be a facilitator and were noted to be related to professional experience. HCPs mentioned that most moments for hand hygiene (e.g., before entering the patient zone) require low cognitive load, but reaching the state of low cognitive investment still requires years of practice. Conversely, non-indicated hand hygiene during moments of pause or when feeling observed was also frequently mentioned in relation to automatisms, then representing a barrier to hand hygiene adherence.	“My goal for it [performing hand hygiene] is to become an automatic process for health care practitioners again, just like they know where the gas and brake pedals are in the car. (…), because having to consciously think about it [performing hand hygiene] absorbs energy and concentration that they [HCPs] need for the patient.” (expert) “[I carried out hand hygiene correctly today] because it has become as routine as brushing my teeth at home. Since I’ve been practicing it [hand hygiene] for so long, I do it [hand hygiene] almost automatically.” (nurse) “It [new hand hygiene technique with forearm disinfection] requires practice because I am used to doing it [hand hygiene technique] differently (…). It took much longer because I always have to remember, oh no, I have to disinfect my forearms first.” (nurse)
	X	Plan workflow	Planning workflows was identified as a facilitator by some experts. They observed that a well-planned sequence of care steps (e.g., preparing materials before entering the patient zone), which is not always practiced by frontline HCPs, leads to a lower number of hand hygiene indications and, consequently, higher adherence. Some frontline staff acknowledged the relationship of a specific workflow and low cognitive load.	“I believe that it [having a workflow] makes [performing hand hygiene] much easier.” (others) “Standardized workflows help to perform correct hand hygiene, but if additional steps or distractions occur, (…) there is a risk [that hand hygiene might not be performed correctly].” (expert)
Physical Opportunity	X	Workload and emergencies	High workload, answering frequent alarms of abnormal vital signs or false alarms from monitored patients, and dealing with emergencies (e.g., apnea episodes in patients) were almost unanimously mentioned as barriers to hand hygiene. Emergencies or perceived emergencies were associated with the active postponement of hand hygiene, as hand hygiene was seen as too time-consuming to safely manage the situation. More experienced HCPs noted that, with increase of professional experience, they became better at correctly assessing situations. Scenarios that they previously deemed incompatible with performing hand hygiene were viewed differently with experience.	“(…) [what prevents me from hand hygiene], are those situations where I have to act quickly and can’t complete the hand disinfection, (…)” (nurse) “(…) [in an emergency situation], you don’t have the time, or you don’t take the time to do it [hand hygiene], the way you really should, (…)” (nurse) “To be honest, I don’t find it entirely realistic to practice proper hand hygiene in our daily routine, because they [the monitored patients] trigger alarms so often, and you have to enter the patient area so frequently, (…).” (nurse) “High workload and staff shortages increase the workload. This combination (…) prevents correct hand hygiene.” (nurse) “You feel like you have to be with every child at the same time, while parents also need something from you. You rush from A to B, and then the alarms need to be answered in between.“ (nurse)
	X	Invisibility of germs	The “invisibility of germs” was rarely mentioned by frontline HCPs, but experts identified this natural fact as a barrier to hand hygiene adherence, as the visual feedback of mistakes made is lacking.	“I believe the difficulty [of performing hand hygiene correctly] lies in the fact that you don’t always see the consequences of missing hand disinfection.” (expert) “[Hand hygiene is] super important to avoid carrying bacteria, viruses, and pathogens from one patient to the next. [You need] to be sensitive to this invisible possibility that you might transmit pathogens.” (others)
	–	Availability of hand disinfectant	The availability of hand disinfectant, a facilitator, was deemed sufficient due to both mounted and wearable dispensers. However, the abundance of hand disinfectant was occasionally perceived as encouraging hand hygiene even when it was not indicated.	“The hand sanitizer dispenser [facilitate hand hygiene] because they are placed in highly visible locations or where they are truly needed, such as where medications are prepared or within the patient area. I find this very convenient. I believe we are exceptionally well-equipped in this regard.” (nurse)
Social Opportunity	X	Having or being a role-model	Both being a role model from the perspective of professional trainers and having a role model from the perspective of apprentices or peers were identified as facilitators for hand hygiene. The presence of role models was predominantly reported by the nursing staff. Conversely, the absence of role models was highlighted by nurses and trainees perceiving (mostly senior) physicians as not adhering to hand hygiene indications.	“I believe that it [hand hygiene] is sometimes done more consciously and carefully when someone is watching me. [As a result] I think twice about whether I have really disinfected my hands thoroughly enough.” (nurse) “(…) but all the senior physicians run past the disinfectant, and the teaching or the ward round begins. I don’t want to be late, so I skip it, even though I actually thought about it [hand hygiene]. If everyone did it [hand hygiene], then we would also have more time [to perform hand hygiene]. That influences me negatively.” (physician) “When I see others not performing [hand hygiene] as they should, then I think - to put it bluntly - why should I do it? Because you [other healthcare practitioner] is not doing it either. And I know no one will accuse me of not performing hand hygiene correctly because they didn’t do it correctly either.” (nurse)
	X	Being observed by others	The mere fact that someone is observing one’s hand hygiene was noted as a strong facilitator. The social pressure to adhere to hand hygiene, coupled with the fear that the observer might detect a moment of non-adherence, was evident. This positive influence applied to various observers, including parents, trainees, IPC team members, peers, and superiors.	“So rather positively [I am influenced when someone observes my hand hygiene performance], I think. Because you know that someone is on the ward who looks more closely [at hand hygiene performance]. Then you do it more thoroughly, of course, like when you might do it alone at night when no one is there.” (physician) “Well, I am more positively influenced [when performing hand hygiene if the parents are watching]. Because you know that the parents are watching and also see that you are correctly disinfecting your hands.” (physician)
Reflective Motivation	X	Experience the consequences of hand hygiene adherence and non-adherence	Recognizing the consequences of adherence or non-adherence of hand hygiene, such as when the ward, patients, and HCPs are affected by a pathogen outbreak or when HCP receive a positive or negative feedback about their own hand hygiene practices, served as a facilitator for adherence. This recognition often prompted the next determinant mentioned, namely self-reflection.	“[The many patients under contact precautions were a key moment for me. It showed me] that you really should take it [hand hygiene] more seriously. That you change a lot with your hands. (…) with the contact precautions, you see the consequences [of inadequate hand hygiene] (…).” (nurse) “Feedback [on hand hygiene performance] is probably one of the best measures [for improving hand hygiene]. It lingers in your memory for quite some time when someone gives you feedback, or in my case criticism and will be longer kept in mind than an email from infection control with three documents attached (…).” (physician)
	X	Self-reflect on one’s hand hygiene behavior	Self-reflecting on one’s behavior, particularly after perceiving the negative consequences of hand hygiene non-adherence, served as a facilitator. This reflection often led to a stronger intention to adhere to hand hygiene (see below).	“When we had to decolonize them [patients colonized with methicillin-resistant *Staphylococcus aureus*], (…) that was a key experience for me because I thought: ‘What a mess, have I always worked hygienically clean and properly and have done correct hand hygiene?’ ”(nurse) “It’s a bit of uncertainty about what the consequence is [if you forget to perform hand hygiene]. You don’t see the germs that you carry from one to another. But when you hear that the next child has tested positive [with a pathogen that was already detected in another patient], I do wonder if I was involved [with inadequate hand hygiene]. That changes something within you.” (nurse)
	X	Intention to adhere to hand hygiene	Having the explicit goal or the intention to adhere to hand hygiene was mentioned as a facilitator for adherence. Several HCPs mentioned that their goal is to perform “perfect” hand hygiene, motivated by the vulnerability of their patients and the professional ethos of “do no harm.”	“I think [hand hygiene] is also part of professional ethics. It has something to do with esteem towards the patient, and I would also like to be treated that way if I were a patient in the hospital.” (physician) “Hand hygiene is important to me and this awareness is imprinted on my mind, it matters greatly to me.” (nurse) “Thinking of the children [helps me perform hand hygiene correctly].” (physician)
	–	Expect hand hygiene to prevent pathogen transmissions	The belief that hand hygiene prevents transmissions was a facilitator frequently acknowledged by frontline staff. It was rarely explicitly mentioned, possibly because the effectiveness of hand hygiene is widely recognized and considered self-evident. When asked directly, however, HCPs expressed confidence that hand disinfection prevents the transmission of pathogens to both patients and them.	“In our field, we deal with patients who have an extremely weak immune system, they have to be carefully protected. That’s why hand hygiene is the most important thing for me because hands are the main carriers of germs.” (nurse) “It [hand hygiene] is about having as few germs as possible on the skin when we interact with our patients who are very susceptible to infections and germs that we as adults are not susceptible to.” (nurse)
	–	(Fear about) Negative consequences from hand hygiene	Negative consequences from hand hygiene, such as skin dryness, was frequently cited, particularly when looking back to the COVID-19 pandemic, when nonstandard hand disinfectant was sometimes used. Nurses commonly expressed concerns about negative effects on patients, such as the possibility that cold hands or alcohol vapors irritate preterm babies. This concern was less frequently mentioned by physicians or experts. Despite that these fears could be acting as barriers, frontline staff reported no impact on hand hygiene adherence: Hands were still disinfected when necessary.	“It could be that [hand hygiene has disadvantages for me]. (…) but in order not to endanger my patients, I simply have to do it.” (nurse) “[As a disadvantage of hand hygiene for the patients, I can imagine] the really cold hands. The disinfectant cools the hands down. (…) and after disinfecting your hands, even when it [the disinfectant] has dried, you just have cool hands, and that is another factor that affects the well-being of babies who are really sensitive to everything, especially when they are newly born. And it doesn’t feel nice to have such cold hands.” (nurse) “I think the only disadvantage [of Hand Hygiene for the patients] is if I approach the children with hands still wet from disinfectant. They look puzzled, inhale the fumes, and I feel like that could be harmful.” (nurse)
	–	Vulnerable patients	Working with vulnerable patients was cited as a significant reason why frontline HCPs strive to adhere to hand hygiene as often as possible. Frontline staff were keenly aware of the vulnerability of their patients and were committed to providing the best care possible, which includes rigorous adherence to hand hygiene practices.	“They [the patients] are immunosuppressed, they are premature, and do not yet have the defenses that adults have. Any skin germs that do not harm us could be very dangerous for them and could cause diseases. Therefore, it is important to disinfect the hands, which touch the patients and other things. To protect them [the patients] as much as possible.” (nurse)
Automatic Motivation	X	Experiencing a guilty conscience when not adhering to hand hygiene	Feeling a guilty conscience when intentionally or unintentionally not adhering to hand hygiene was described as a strong facilitator, as HCPs strive to avoid this negative emotion.	“[When I notice that I forgot to perform hand hygiene], I then think: ‘Oh no, I should have disinfected my hands’. I then have a guilty conscience.” (nurse)

*Abbreviations*: COM-B, Capability, Opportunity, Motivation and Behavior model; HCP, healthcare practitioner; IPC, Infection prevention and control.

Four determinants were found in “psychological Capability,” all of them considered high-priority: “knowledge about indications and technique” of hand hygiene and “habits and automatisms” were mentioned as being present as both facilitator and barrier. The lack of “attention control to perform hand hygiene in daily routine” and “planning of workflows” were described as barriers.

Three determinants were assigned to “physical Opportunity.” “Workload and emergencies” and the inherent “invisibility of germs” were considered high-priority and were mentioned as barriers. The “availability of hand disinfectant” was classified as a non-priority facilitator as hand disinfectant is sufficiently available in the USZ. Two determinants assigned to the “social Opportunity” source, namely “role-models” and “being observed by others,” were deemed high-priority facilitators.

Six determinants were assigned to “reflective Motivation” and three thereof were high-priority facilitators: The “intention to adhere to hand hygiene,” was central, with “perceiving the consequences of (non-)adherence,” and “self-reflect on one’s hand hygiene behavior” that influence adherence via this determinant. “Expect hand hygiene to prevent transmissions” was considered a non-priority facilitator as it was deemed so obvious that no further addressing was necessary. The same was true for caring for “vulnerable patients,” a strong facilitator for adherence. The barrier “negative consequences from hand hygiene,” like dryness of hands, has improved after return to the usual hand hygiene product that was temporarily replaced during the COVID-19 pandemic and thus deemed non-priority. The only high-priority facilitator that was assigned to “automatic Motivation” was “having a bad conscience” when not adhering to hand hygiene.

## Discussion

In this paper, we report the findings of an in-depth and theory-based analysis of the behavioral determinants of hand hygiene adherence in neonatology. With this qualitative study, which allows a deeper understanding of HCP perceptions and drivers compared to quantitative methods, we identified 16 determinants. Twelve were considered of high priority to increase hand hygiene adherence in our setting. Four high-priority determinants were identified in each of the COM-B domains “psychological Capability,” “environmental and social Opportunity,” and “reflective and automatic Motivation.” While themes from “Capability” and “Opportunity” were both facilitators and barriers, high-priority “motivational” determinants exclusively were facilitators.

Several other study groups have investigated drivers for (non-)adherence in hand hygiene, some in neonatology,^2,18^ but the majority in non-neonatal settings. Similar to the present study, most investigations identified a range of barriers and facilitators rather than just one or a few.^19,20^ Fuller et al., who investigated reasons for hand hygiene non-adherence on ICUs and acute care units by interrogating HCP immediately after hand hygiene observation, identified “memory/attention/decision making” and “knowledge” as the main determinants for hand hygiene noncompliance.^21^ Similarly, Pasricha et al. who assessed barriers through questionnaire and thus with a greater distance from the action, identified “forgetfulness,” “lack of awareness,” and “lack of knowledge.”^2^ Such determinants categorized under the COM-B “Capability” domain, are per definition intrinsic to HCPs, and were confirmed by several other authors.^22–25^ While our interviewees also mentioned some of these as barriers (i.e., “lack of attention control” or “planning of workflows”), initially they expressed confidence in their knowledge and ability to perform hand hygiene. However, upon further questioning, some acknowledged knowledge gaps. On the contrary, the IPC experts, who were conducting hand hygiene interventions and adherence observations in the past, mentioned “knowledge” as one of the most common barriers in frontline staff. This could be attributed to individuals either not recognizing their own knowledge gaps or feeling hesitant to admit them in an interview situation. While “knowledge,” “attention control,” “planning of workflow” sure all are important for hand hygiene adherence, they all require active investment of HCP. Ideally, hand hygiene is performed automatically as a habitual behavior. As such, hand hygiene habits developed through years of work experience were often cited as facilitators.

The most frequently mentioned determinants in our study were those lying outside of the HCP’s perceived own influence, in the COM-B “Opportunity” domain. The high workload and patient emergency situations were often cited as barriers. This appears to be a common hindering factor in various hospital settings worldwide,^2,3,22,26–28^ likely linked to the high cognitive load, distraction, and competing priorities such situations bring along. On the other hand, the “availability of hand disinfectant” was perceived to be sufficiently present in our setting and thus was seen as non-priority determinant. In other hospitals, both in resource-limited countries, where there might be fewer hand disinfectant dispensers, and in higher resource settings, where dispensers were e.g., reported to be empty, non-availability of hand disinfectant was identified as one of the most relevant barriers.^26,29–31^ In our neonatal setting, social influences, such as “having role models” or “being observed by others,” particularly parents, were important facilitators. This was also shown in an interview study with HCPs from ICUs that describes the sense of “being watched” and “reminders and encouragement from peers” to improve adherence,^28^ and a questionnaire study that found that the “opinion of important others” influences hand hygiene behavior.^18^ These findings encourage the use of peer feedback and the fostering of a speak-up culture.

While our study identified several determinants in the COM-B “reflective and automatic Motivation” domain, such determinants were only rarely reported by other authors.^28,32^ We hypothesize that the relevance of the motivational determinants is linked to the neonatal setting where patients are highly vulnerable and dependent, and the frontline staff feel a particularly high obligation to protect them from adverse events. Additionally, in neonatology, pathogen transmission events are frequently detected due to high microbiological screening activity. The determinants we identified in the “motivational” domain included active cognitive processes and reflections that – similar to the behavioral model of the “theory of planned behavior”^33^ – all ultimately influenced the “intention or goal to adhere” to hand hygiene. Most motivational determinants were positive influencers, leading to an increase of the “intention to do hand hygiene.” However, how much the “intention to perform hand hygiene” predicts actual hand hygiene adherence is unclear, and was questioned by O’Boyle et al., who postulated that hand hygiene behavior may be more sensitive to external factors than to internal motivational factors.^34^

As several other study groups, we perceived the use of the TDF to guide our analysis as useful.^20,21^ Assigning the determinant themes also to the COM-B model will later facilitate the tailoring of implementation strategies using the Behavior Change Wheel, a comprehensive framework that guides the design of behavior change interventions. In the process of introducing behavior change, prioritization of determinants – i.e., identifying important determinants – is one of the crucial steps to be carried out. In our setting, four determinants were indeed found but their addressing was perceived not necessary, either because the concepts or circumstances were well understood, obvious, or had already been addressed in the past. While “importance” is one prioritization criterion, “changeability” – the ease or difficulty of changing a factor – could be another.^35^ Highly changeable determinants, often termed as low-hanging fruits, could be initially targeted in behavior change interventions, especially when they are also considered important. Notably, not only the determinants themselves but also their classification based on importance and changeability can vary across settings, based upon specific contexts and priorities.

While this study has several strengths such as the theory-based approach, that facilitated comparison of our results with other papers, and the cumulative 16 hours of interviews with purposefully selected HCP, it also has some limitations. First, as in all interview studies, a social desirability bias cannot be excluded. Interviewees might have answered the questions in a way they believed to be more acceptable or favorable rather than expressing their true thoughts and behaviors. To mitigate this potential bias, we selected an interviewer who was not part of the IPC or the neonatology team, and who made efforts to create an atmosphere of openness and trust. We also included IPC experts as interview partners, who, based on their experience and close contact with frontline HCP, were able to provide a likely more objective perspective on the frontline staff’s hand hygiene behavior. Second, there is a risk of selection bias, as the interviews were conducted on a voluntary basis and employees with a positive attitude towards hand hygiene might have been more likely to participate. Third, the interviews were conducted in German, which might have led to employees with German as a second language being less likely to participate, potentially leading to reduced diversity of interviewees. Lastly, this was a single-center study conducted in a high-resource setting, and the findings may thus not be transferable to all other settings.

In conclusion, this qualitative interview study identified several determinants influencing hand hygiene adherence in a neonatology setting. Some are well-known, lying inside an individual HCP such as knowledge about indications or automatisms, or outside of HCP such as high workload and being watched by others. Others are new and likely particularly specific for the neonatal setting, such as the motivational determinants of self-reflection on hand hygiene behavior or the very strong intention to adhere to hand hygiene. Our findings can now inform hand hygiene interventions that are tailored to the needs of HCPs in the neonatology wards, optimizing the use of time and personnel resources. A study comparing the determinants of hand hygiene adherence in both adult ICUs and NICUs is needed to confirm and further elucidate the hypothesized differences between these settings.

## Supporting information

Bopp et al. supplementary material 1 Bopp et al. supplementary material

Bopp et al. supplementary material 2 Bopp et al. supplementary material

Bopp et al. supplementary material 3 Bopp et al. supplementary material

Bopp et al. supplementary material 4 Bopp et al. supplementary material

Bopp et al. supplementary material 5 Bopp et al. supplementary material

## Supplementary material

To view supplementary material for this article, please visit https://doi.org/10.1017/ice.2025.82

## References

[1] (WHO) WHO. SAVE LIVES Clean your hands: Guide to Implementation 2009; https://www.who.int/publications/i/item/a-guide-to-the-implementation-of-the-who-multimodal-hand-hygiene-improvement-strategy. Accessed 19.07.2024.

[2] Pasricha SV , Singh M , Pasricha R , Jimal D , Khurshid F. Neonatal intensive care unit hand hygiene: exploring current practice and adherence barriers in a Canadian hospital. Can J Infect Control 2021;36:77–85.

[3] Song X , Stockwell DC , Floyd T , Short BL , Singh N. Improving hand hygiene compliance in health care workers: strategies and impact on patient outcomes. Am J Infect Control. 2013;41:e101–105. 23643451 10.1016/j.ajic.2013.01.031

[4] Lambe KA , Lydon S , Madden C , et al. Hand hygiene compliance in the ICU: a systematic review. Crit Care Med. 2019;47:1251–1257. 31219838 10.1097/CCM.0000000000003868

[5] Erasmus V , Daha TJ , Brug H , et al. Systematic review of studies on compliance with hand hygiene guidelines in hospital care. Infect Control Hosp Epidemiol. 2010;31:283–294. 20088678 10.1086/650451

[6] Wolfensberger A , Schmid M , Sax H , et al. Determinants for voluntary participation in staff screening during an methicillin-resistant *Staphylococcus aureus* (MRSA) outbreak on a neonatal ward. Infect Control Hosp Epidemiol. 2021;42:881–884. 33256866 10.1017/ice.2020.1319

[7] Achermann Y , Seidl K , Kuster SP , et al. Epidemiology of methicillin-susceptible *Staphylococcus aureus* in a neonatology ward. Infect Control Hosp Epidemiol. 2015;36:1305–1312. 26290400 10.1017/ice.2015.184

[8] Hosoglu S , Hascuhadar M , Yasar E , Uslu S , Aldudak B. Control of an Acinetobacter [corrected] baumannii outbreak in a neonatal ICU without suspension of service: a devastating outbreak in Diyarbakir, Turkey. Infection. 2012;40:11–18. 21881956 10.1007/s15010-011-0180-y

[9] Michie S , van Stralen MM , West R. The behaviour change wheel: a new method for characterising and designing behaviour change interventions. Implement Sci. 2011;6:42. 21513547 10.1186/1748-5908-6-42 PMC3096582

[10] Michie S , Johnston M , Abraham C , et al. Making psychological theory useful for implementing evidence based practice: a consensus approach. Qual Saf Health Care. 2005;14:26–33. 15692000 10.1136/qshc.2004.011155 PMC1743963

[11] Cane J , O’Connor D , Michie S. Validation of the theoretical domains framework for use in behaviour change and implementation research. Implement Sci. 2012;7:37. 22530986 10.1186/1748-5908-7-37 PMC3483008

[12] Baker R , Camosso-Stefinovic J , Gillies C , et al. Tailored interventions to address determinants of practice. Cochrane Database Syst Rev. 2015;2015:CD005470. 25923419 10.1002/14651858.CD005470.pub3 PMC7271646

[13] Nyantakyi E , Caci L , Castro M , et al. Implementation of infection prevention and control for hospitalized neonates: a narrative review. Clin Microbiol Infect. 2024;30:44–50. 36414203 10.1016/j.cmi.2022.11.007

[14] Douglas S , Cartmill R , Brown R , et al. The work of adult and pediatric intensive care unit nurses. Nurs Res. 2013;62:50–58. 23222843 10.1097/NNR.0b013e318270714b PMC5890532

[15] van Zanten HA , Tan RN , Thio M , et al. The risk for hyperoxaemia after apnoea, bradycardia and hypoxaemia in preterm infants. Arch Dis Child Fetal Neonatal Ed. 2014;99:F269–273. 24668832 10.1136/archdischild-2013-305745

[16] Patton M. Qualitative research & evaluation methods. Thousand Oaks, 3rd ed. CA: Sage Publications; 2002:541–588.

[17] StataCorp. Stata Statistical Software: Release 16-1. College Station, TX: StataCorp LLC. 2023.

[18] Pessoa-Silva CP-B, K. ; Pfister, R. ; Touveneau, S. ; Perneger, TV. ; Pittet, D. Attitudes and perceptions toward hand hygiene among healthcare workers caring for critically ill neonates. Infect Control Hosp Epidemiol. 2005;26:305–311. 15796285 10.1086/502544

[19] Boscart VM , Fernie GR , Lee JH , Jaglal SB. Using psychological theory to inform methods to optimize the implementation of a hand hygiene intervention. Implement Sci. 2012;7:77. 22929925 10.1186/1748-5908-7-77 PMC3503739

[20] Yehouenou CL , Abedinzadeh A , Houngnihin R , et al. Understanding Hand Hygiene behavior in a public hospital in benin using the theoretical domain frameworks: the first step for designing appropriate interventions. Healthcare (Basel). 2022;10. 10.3390/healthcare10101924 PMC9602033 36292370

[21] Fuller C , Besser S , Savage J , McAteer J , Stone S , Michie S. Application of a theoretical framework for behavior change to hospital workers’ real-time explanations for noncompliance with hand hygiene guidelines. Am J Infect Control. 2014;42:106–110. 24355490 10.1016/j.ajic.2013.07.019

[22] Dyson JL, R. ; Jackson, C. ; Cheater, F. Development of a theory-based instrument to identify barriers and levers to best hand hygiene practice among healthcare practitioners. Implement Sci. 2013;23;:111. 10.1186/1748-5908-8-111 PMC3850814 24059289

[23] Huis A , van Achterberg T , de Bruin M , Grol R , Schoonhoven L , Hulscher M. A systematic review of hand hygiene improvement strategies: a behavioural approach. Implement Sci. 2012;7:92. 22978722 10.1186/1748-5908-7-92 PMC3517511

[24] Mathur P. Hand hygiene: back to the basics of infection control. Indian J Med Res. 2011;134:611–620. 22199099 10.4103/0971-5916.90985 PMC3249958

[25] Le CD , Lehman EB , Nguyen TH , Craig TJ. Hand hygiene compliance study at a large central hospital in Vietnam. Int J Environ Res Public Health. 2019;16. 10.3390/ijerph16040607 PMC6406810 30791457

[26] Deshommes T , Nagel C , Tucker R , et al. A quality improvement initiative to increase hand hygiene awareness and compliance in a neonatal intensive care unit in Haiti. J Trop Pediatr. 2021;67. 10.1093/tropej/fmaa029 32594158

[27] Amaan A , Dey SK , Zahan K. Improvement of hand hygiene practices among the healthcare workers in a neonatal intensive care unit. Can J Infect Dis Med Microbiol. 2022;2022:7688778. 35795864 10.1155/2022/7688778 PMC9252715

[28] Lambe K , Lydon S , Madden C , et al. Understanding hand hygiene behaviour in the intensive care unit to inform interventions: an interview study. BMC Health Serv Res. 2020;20:353. 32334574 10.1186/s12913-020-05215-4 PMC7183607

[29] Bezerra TB , Valim MD , Bortolini J , et al. Influencing factors of hand hygiene in critical sections of a Brazilian hospital. J Infect Dev Ctries. 2021;15:840–846. 34242195 10.3855/jidc.13658

[30] Kumar A , Kumar R , Gupta AK , et al. Improvement of hand hygiene compliance using the plan-do-study-act method: quality improvement project from a tertiary care institute in Bihar, India. Cureus. 2022;14:e25590. 35664291 10.7759/cureus.25590 PMC9162031

[31] Vaughan-Malloy ACY , Sandora TJ. Using a human factors framework to assess clinician perceptions of and barriers to high reliability in hand hygiene. Am J Infect Control 2023;51:514–519. 36933570 10.1016/j.ajic.2023.01.013

[32] Squires JE , Linklater S , Grimshaw JM , et al. Understanding practice: factors that influence physician hand hygiene compliance. Infect Control Hosp Epidemiol. 2014;35:1511–1520. 25419774 10.1086/678597

[33] Ajzen I. The theory of planned behavior. Organ Behav Hum 1991;50:179–211.

[34] O’Boyle CA , Henly SJ , Larson E. Understanding adherence to hand hygiene recommendations: the theory of planned behavior. Am J Infect Control. 2001;29:352–360. 11743481 10.1067/mic.2001.18405

[35] Green LW , Gielen AC , Peterson DV , Kreuter MW , Ottoson JM. Health Program Planning, Implementation, and Evaluation. Johns Hopkins University Press; 2022.

