# Supplementary material for: Understanding hand hygiene adherence in neonatology: a qualitative study of behavioral determinants
Source: Infect Control Hosp Epidemiol. 2025 May 16;46(7):738–46. doi: 10.1017/ice.2025.82 (PMC12277078; doi:10.1017/ice.2025.82)
Supplement: Bopp et al. supplementary material 1 — Bopp et al. supplementary material [file S0899823X25000820sup001.docx]

# **Appendix Table 1 The ‘four moments for hand hygiene’ in the USZ neonatology ward**

| **4 Moments for Hand Hygiene** | **Additional Forearm Disinfection** |
| --- | --- |
| Entering the patient zone* |  |
| Before patient contact | X |
| Before a clean/aseptic procedure |  |
| After contact with body fluids |  |
| Leaving the patient zone* |  |
| After patient contact | X |

**Caption**: Table summarizing the concept of the ‘four moments for hand hygiene’, followed by the USZ neonatology ward. It is an adapted version of the ‘WHO my five moments for hand hygiene’ ^1^. The four Moments for hand hygiene are presented in the first column, while the second columns use an X to indicate if an additional forearm disinfection is required. *The patient zone for each individual patient is either defined by a physical single room or by a visible floor marking. It includes not only the patient’s bed but also necessary equipment (e.g., respirator or monitoring devices) and the patient’s belongings. To reduce the need for frequent hand hygiene due to repeated entries into the patient zones, a remote control for silencing vital parameter alarms has been placed outside each patient zone.

**Abbreviations:** USZ, University Hospital Zurich
