# Supplementary material for: Understanding hand hygiene adherence in neonatology: a qualitative study of behavioral determinants
Source: Infect Control Hosp Epidemiol. 2025 May 16;46(7):738–46. doi: 10.1017/ice.2025.82 (PMC12277078; doi:10.1017/ice.2025.82)
Supplement: Bopp et al. supplementary material 2 — Bopp et al. supplementary material [file S0899823X25000820sup002.docx]

# Appendix 2 The domains of the TDF with in-detail description and their mapping to COM-B components according to Cane et al.

| **COM-B Component** | **TDF Domain** | **TDF Description** |
| --- | --- | --- |
| **Psychological Capability^+^**  The psychological capacity to engage in the activity concerned. | Knowledge | An awareness of the existence of something. |
|  | Skills | An ability or proficiency acquired through practice. |
|  | Memory, Attention, and Decision Processes | The ability to retain information, focus selectively on aspects of the environment, and choose between two or more alternatives. |
|  | Behavioral Regulation | Anything aimed at managing or changing objectively observed or measured actions. |
| **Physical Opportunity**  The physical factors outside of the individual that prompt the behavior. | Environmental Context and Resources | Any circumstance of a person's situation or environment that discourages or encourages the development of skills and abilities, independence, social competence, and adaptive behavior. |
| **Social Opportunity**  The social factors outside of the individual that prompt the behavior. | Social Influences | Those interpersonal processes that can cause individuals to change their thoughts, feelings, or behaviors. |
| **Reflective Motivation**  The motivation of the analytical brain process that shapes the individual. | Social/Professional Role and Identity | A coherent set of behaviors and displayed personal qualities of an individual in a social or work setting. |
|  | Beliefs about capability | Acceptance of the truth, reality, or validity about an ability, talent, or facility that a person can put to constructive use. |
|  | Optimism | The confidence that things will happen for the best or that desired goals will be attained. |
|  | Beliefs about Consequences | Acceptance of the truth, reality, or validity about outcomes of a behavior in a given situation. |
|  | Goal ^*)^ | Increasing the probability of a response by arranging a dependent relationship, or contingency, between the response and a given stimulus. |
|  | Intentions ^*)^ | A conscious decision to perform a behavior or a resolve to act in a certain way. |
| **Automatic Motivation**  The motivation of the emotional/habitual brain process that shapes the individual. | Reinforcement | Mental representations of outcomes or end states that an individual wants to achieve. |
|  | Emotion | A complex reaction pattern, involving experiential, behavioral, and physiological elements, by which the individual attempts to deal with a personally significant matter or event. |

**Caption**: In-detail description of the domains of the TDF according to Cane et al. ^10,11^ and their mapping to the Behavioral Change Wheel's COM-B components by Michie et al. ^9^ The COM-B model consists of three domains, each composed of two-subdomains, which interact to generate behavior and can be broken down more granularly in the domains of the TDF. The COM-B model forms the central hub of the behavior change wheel, that provides a structured approach for designing interventions. The TDF provides a structured and multidisciplinary method to identify and analyze factors influencing behavior and behavior change. It integrates theories from various disciplines including psychology, sociology and behavioral science and provides a comprehensive and broad understanding of behavior and behavior change.

*) Goals and intentions were merged. **^+^**) The subdomain Physical Capability is not shown as it is not relevant for our discussion since every participant in our study is physically able to perform Hand Hygiene.

**Abbreviations**: COM-B, Capability, Opportunity, Motivation and Behavior model; TDF, Theoretical domains framework
