# Supplementary material for: Understanding hand hygiene adherence in neonatology: a qualitative study of behavioral determinants
Source: Infect Control Hosp Epidemiol. 2025 May 16;46(7):738–46. doi: 10.1017/ice.2025.82 (PMC12277078; doi:10.1017/ice.2025.82)
Supplement: Bopp et al. supplementary material 3 — Bopp et al. supplementary material [file S0899823X25000820sup003.docx]

# Appendix 3 Interview Guide

| **Main Questions** | **Follow-up questions** |
| --- | --- |
| Can you name two terms that come to your mind when you think about hand hygiene? | How do you come up with these terms? |
| What motivated you to perform hand hygiene this morning?  Why did you perform hand hygiene this morning? | How important is hand hygiene in your profession?  Why is it important for the patients that you perform hand hygiene correctly?  Can you imagine any disadvantages of hand hygiene for the patients?  Are there also benefits for you when you perform hand hygiene?  Are there also disadvantages for you when you perform hand hygiene? |
| How many of the necessary hand hygiene can you perform correctly on average? Try to estimate a percentage | How do you achieve such a percentage of correct hand hygiene?  What circumstances or situations make it difficult for you to perform hand hygiene?  Is hand hygiene an automatic action for you or do you need to remind yourself actively?  If it's an active reminder: What would help you to remember to perform hand hygiene? Have you discovered anything that helps you? |
| What measures have been implemented in the neonatology unit to facilitate hand hygiene adherence? | Which measures do you find helpful?  Which measures do you find unnecessary or confusing?  Have there been key moments in the past that have helped you improve your hand hygiene? |
| How are you influenced by others in your hand hygiene behavior? | How are you influenced by colleagues, supervisors, or parents in your hand hygiene behavior?  Do you receive appreciation when you perform hand hygiene correctly?  Do you receive criticism when you perform hand hygiene incorrectly? |
| Imagine you just forgot to perform hand hygiene. How does that make you feel? |  |
| Do you know the correct technique for performing hand hygiene? |  |
| Do you know the correct indications for performing hand hygiene? |  |
| Has it ever happened to you that you were unsure whether you needed to perform hand hygiene? | In which situations or for what reasons were you unsure? |
| When you have questions about hand hygiene, where do you seek your answers? |  |
| If you had to plan measures yourself to achieve 100% correct hand hygiene, which measures would you choose? |  |
| How old are you? |  |
| What is your gender? |  |
| What is your position in the neonatology unit? |  |
| How long have you been working in this profession/when did your training start? How long have you been working in neonatology/for the neonatology unit? |  |

**Caption:** Interview Guide used for the interviews. These questions were also directed to the experts, but they were instructed to take a third person’s perspective and not to answer for themselves, but rather to provide an assessment of the frontline health care professional
