# Supplementary material for: Understanding hand hygiene adherence in neonatology: a qualitative study of behavioral determinants
Source: Infect Control Hosp Epidemiol. 2025 May 16;46(7):738–46. doi: 10.1017/ice.2025.82 (PMC12277078; doi:10.1017/ice.2025.82)
Supplement: Bopp et al. supplementary material 4 — Bopp et al. supplementary material [file S0899823X25000820sup004.docx]

# Appendix 4 Frequency of barriers and facilitators according to Theoretical Domains Framework (TDF) domain

| COM-B | TDF-Domain | Segments coded as barriers n (% of all barriers) | Segments coded as facilitators n (% of all facilitators) | Segments coded as either barriers or facilitators n (% of all segments) |
| --- | --- | --- | --- | --- |
| Psychological Capability | Knowledge | 55 (9.4) | 35 (4.1) | 90 (6.2) |
|  | Skills | 22 (3.7) | 28 (3.3) | 50 (3.5) |
|  | Memory, attention and decision process | 121 (20.6) | 60 (7.0) | 181 (12.5) |
|  | Behavioral regulations | 1 (0.2) | 28 (3.3) | 29 (2.0) |
| Physical Opportunity | Environmental context and resources | 174 (29.6) | 313 (36.4) | 487(33.7) |
| Social Opportunity | Social influences | 34 (5.8) | 76 (8.8) | 110 (7.6) |
| Reflective Motivation | Social / professional role and identity | 9 (1.5) | 43 (5.0) | 52 (3.6) |
|  | Beliefs about capability | 20 (3.4) | 15 (1.7) | 35 (2.4) |
|  | Optimism | 10 (1.7) | 1 (0.1) | 11 (0.8) |
|  | Beliefs about consequences | 110 (18.7) | 91 (10.6) | 201 (13.9) |
|  | Goals & Intentions | 3 (0.5) | 66 (7.7) | 69 (4.8) |
| Automatic Motivation | Reinforcement | 12 (2.0) | 62 (7.2) | 74 (5.1) |
|  | Emotions | 16 (2.7) | 42 (4.9) | 58 (4.0) |
| Total |  | 587 (40.6) | 860 (59.4) | 1447 (100) |

**Caption**: Number of interview segments assigned to the specific domains of the TDF, grouped in barriers, facilitators and both.

**Abbreviations**: COM-B, Capability, Opportunity, Motivation and Behavior model; TDF, Theoretical domains framework
