# Supplementary material for: Understanding hand hygiene adherence in neonatology: a qualitative study of behavioral determinants
Source: Infect Control Hosp Epidemiol. 2025 May 16;46(7):738–46. doi: 10.1017/ice.2025.82 (PMC12277078; doi:10.1017/ice.2025.82)
Supplement: Bopp et al. supplementary material 5 — Bopp et al. supplementary material [file S0899823X25000820sup005.docx]

# Appendix 5 Frequencies of determinants according to professional groups

| **TDF Domain** | **Nurses n (%)** | | |  | | **Physicians n (%)** | | |  | | **Other professional groups n (%)** | | |  | | **Experts n (%)** | | |
| --- | --- | --- | --- | --- | --- | --- | --- | --- | --- | --- | --- | --- | --- | --- | --- | --- | --- | --- |
|  | **B** | **F** | **Total** | |  | **B** | **F** | **Total** | |  | **B** | **F** | **Total** | |  | **B** | **F** | **Total** |
| **Knowledge** | 27 (7.7) | 24 (4.3) | 51 (5.6) | |  | 7 (8.4) | 2 (2.0) | 9 (4.9) | |  | 9 (14.3) | 5 (4.2) | 14 (7.7) | |  | 12 (13.2) | 4 (5.2) | 16 (9.5) |
| **Skills** | 9 (2.6) | 18 (3.2) | 27 (3.0) | |  | 2 (2.4) | 2 (2.0) | 4 (2.2) | |  | 3 (4.8) | 6 (5.1) | 9 (5.0) | |  | 8 (8.8) | 2 (2.6) | 10 (6.0) |
| **Social / professional role and identity** | 2 (0.6) | 28 (5.0) | 30 (3.3) | |  | 5 (6.0) | 6 (5.9) | 11 (5.9) | |  | 0 | 5 (4.2) | 5 2.8) | |  | 2 (2.2) | 4 (5.2) | 6 (3.6) |
| **Beliefs about capability** | 10 (2.9) | 9 (1.6) | 19 (2.1) | |  | 6 (7.2) | 0 | 6 (3.2) | |  | 0 | 4 (3.4) | 4 (2.2) | |  | 4 (4.4) | 2 (2.6) | 6 (3.6) |
| **Optimism** | 8 (2.3) | 1 (0.2) | 9 (1.0) | |  | 2 (2.4) | 0 | 2 (1.1) | |  | 0 | 0 | 0 | |  | 0 | 0 | 0 |
| **Beliefs about consequences** | 76 (21.7) | 69 (12.3) | 145 (15.9) | |  | 19 (22.9) | 7 (6.9) | 26 (14.1) | |  | 11 (17.5) | 14 (11.9) | 25 (13.8) | |  | 4 (4.4) | 1 (1.3) | 5 (3.0) |
| **Reinforcement** | 8 (2.3) | 39 (6.9) | 47 (5.1) | |  | 2 (2.4) | 9 (8.8) | 11 (5.9) | |  | 1 (1.6) | 7 (5.9) | 8 (4.4) | |  | 1 (1.1) | 7 (9.1) | 8 (4.8) |
| **Goals** | 2 (0.6) | 39 (6.9) | 41 (4.5) | |  | 1 (1.2) | 10 (9.8) | 11 (5.9) | |  | 0 | 12 (10.2) | 12 (6.6) | |  | 0 | 5 (6.5) | 5 (3.0) |
| **Memory, attention and decision process** | 72 (20.6) | 44 (7.8) | 116 (12.7) | |  | 10 (12.0) | 6 (5.9) | 16 (8.6) | |  | 13 (20.6) | 5 (4.2) | 18 (9.9) | |  | 26 (28.6) | 5 (6.5) | 31 (18.5) |
| **Environmental context and resources** | 106 (30.3) | 200 (35.5) | 306 (33.5) | |  | 24 (28.9) | 38 (37.3) | 62 (33.5) | |  | 17 (27.0) | 36 (30.5) | 53 (29.3) | |  | 27 (29.7) | 39 (50.6) | 66 (39.3) |
| **Social influences** | 21 (6.0) | 48 (8.5) | 69 (7.6) | |  | 4 (4.8) | 12 (11.8) | 16 (8.6) | |  | 4 (6.3) | 9 (7.6) | 13 (7.2) | |  | 5 (5.5) | 7 (9.1) | 12 (7.1) |
| **Emotions** | 9 (2.6) | 26 (4.6) | 35 (3.8) | |  | 1 (1.2) | 8 (7.8) | 9 (4.9) | |  | 5 (7.9) | 8 (6.8) | 13 (7.2) | |  | 1 (1.1) | 0 | 1 (0.6) |
| **Behavioral regulations** | 0 | 18 (3.2) | 18 (2.0) | |  | 0 | 2 (2.0) | 2 (1.1) | |  | 0 | 7 (5.9) | 7 (3.9) | |  | 1 (1.1) | 1 (1.3) | 2 (1.2) |
| **Total** | **350 (100)** | **563 (100)** | **913 (100)** | |  | **83 (100)** | **102 (100)** | **185 (100)** | |  | **63 (100)** | **118 (100)** | **181 (100)** | |  | **91 (100)** | **77 (100)** | **168 (100)** |

**Caption**: Frequencies between the professional groups were compared in the top three commonly mentioned determinants by a two sampled test of proportions. The only statistically significant difference (p<0.05) was seen in ‘beliefs about consequences’ between experts and the three other professional groups (data not shown in detail).

**Abbreviations**: TDF, Theoretical domains framework
